# An Interwoven Psychological Syndrome of Job Burnout and Work Engagement in Construction Project Management Professionals Due to Work–Family Imbalance

**DOI:** 10.3390/ijerph192114111

**Published:** 2022-10-28

**Authors:** Xiaodong Li, Runshuang Wang, Yizhu Zhao, Fan Yang, Xinyi Wang

**Affiliations:** 1 Department of Construction Management, School of Civil Engineering, Tsinghua University, Beijing 100084, China; 2 School of Management Science & Engineering, Central University of Finance and Economics, Beijing 100081, China

**Keywords:** construction project management professionals, work–family imbalance, job burnout, work engagement, family-supportive supervisor behavior

## Abstract

Most current studies on the mental health of construction project management professionals (CPMPs) are conducted from a negative psychological perspective, lacking a comprehensive understanding of the positive–negative interwoven mechanism. This study developed a positive–negative dual-process psychological model of CPMPs to explore the interwoven mechanisms among five variables: family-supportive supervisor behavior (FSSB), work–family conflict, work–family enrichment, job burnout, and work engagement. We conducted a large-scale questionnaire survey among Chinese CPMPs. A total of 656 questionnaires were returned; 446 were considered valid. The groups of CPMPs prone to occupational psychological problems were identified, which enhanced the targeted organizational management in the construction industry. The hypothetical model was verified with SEM. The results revealed that the effect of work–family enrichment was more significant than work–family conflict, which implies that the positive psychology process may play a more prominent role than the negative process. There was a significant correlation between FSSB and work–family conflict/ enrichment; but no direct correlation between FSSB and job burnout/work engagement. This implies that the improvement of the work–family relationship plays a full mediating role in improving CPMPs’ occupational psychological health. This research provides a thorough understanding of CPMPs’ interwoven occupational psychological problems and gives suggestions to enhance their occupational psychological health.

## 1. Introduction

When scholars focus on the occupational mental health of construction project management professionals (CPMPs) on site, they generally explain the influence mechanisms of occupational mental health from the perspective of negative effects, but we often ignore the positive effects in occupational health psychology. For instance, studies on the work–family relationship have primarily examined the conflicts that arise between work and family, and studies on work status have mostly explored the generation and impact of job stress and burnout [1,2].

The current research perspective reflects the inherent characteristics of the construction management industry, such as an unstable workplace, irregular working hours [3], serious conflicts in coordinating resources and benefits, and the separation of enterprise and project organizations, which tend to cause a series of negative mental health problems [4,5,6]. Such negative psychology may lead to multiple chronic effects on individuals, families, projects, organizations, and society [7]. Studying these negative psychological effects provides theoretical support for understanding the negative psychological generation and action mechanisms. It establishes a foundation for improving project professionals’ occupational mental health status to a certain extent.

Instead of looking exclusively at the negative side, researchers extended their interest to the positive side of worker’s well-being [8]. This shift reflects an emerging trend toward positive psychology, focusing on human strengths and optimal functioning rather than on weaknesses and malfunctioning [9]. Positive psychology integrates the research paradigm of negative psychology, allowing us to understand the mechanisms of occupational health psychology from a new angle [10]. Further, it complements the field of traditional negative psychology, rather than being its antithesis [11]. There is not necessarily a negative correlation between the two. Based on secondary analyses of earlier studies, Russell and Carroll showed convincingly that positive and negative affect are independent states [12], rather than two opposite poles of the same bipolar dimension [13]. For example, job burnout in negative psychology and work engagement in positive psychology do not form a pair of mutually exclusive mental health states [8,14]. An employee can experience job burnout and work engagement due to stressful work and heavy family responsibilities. Thus, although a great deal of negative psychological research was conducted in construction management, it still does not give us a clear depiction of the psychological states of professionals.

George suggested that rather than studying the positive or negative effects in a separate or piecemeal fashion, a more veridical account of the role of affect in organizations might be obtained by considering their combined effects [15]. Positive and negative psychological methods can build a comprehensive analytical framework for occupational mental health, thereby fostering a systematic understanding of CPMPs’ interwoven mental health mechanisms. Therefore, the introduction of the positive psychology perspective may provide a reference to construct a dual path analysis framework for CPMPs to comprehensively understand the causes and effects of mental health.

Organizational support was the first thought in many studies on revealing how to improve work–family relationships and occupational mental health [1]. However, supervisors have considerable discretion over the types and level of support that employees receive, irrespective of whether family-friendly benefits are provided by the organization [16,17]. Therefore, family supportive supervision has emerged as an important prerequisite for effective work–family integration and employees’ well-being [18]. Hammer argued that most measures of family-supportive supervision and general supervisor support were based primarily on emotional support dimensions, and it is important to more clearly conceptualize family-supportive supervision by identifying specific behaviors that supervisors enact [19], hence, the concept of family-supportive supervisor behavior (FSSB) was proposed. Previous studies have examined the outcomes of FSSB, including work–family outcomes, work outcomes, and health outcomes [20,21,22,23]. The current status of FSSB among CPMPs and its impact on occupational mental health in the construction industry remains understudied.

Based on the current situation of research and practice, this study developed a positive–negative dual-process psychological model rooted in retaining the traditional negative psychological effects to reflect the actual interwoven psychological process comprehensively. Hence, this study examined the positive effects of work–family enrichment and work engagement, together with the traditional work–family conflict and job burnout from the negative perspective. Through a large-scale survey, this study also investigated FSSB of Chinese CPMPs to reveal their current situation and influential effects of occupational mental health.

## 2. Literature Review

### 2.1. Research on Work–Family Conflict and Work–Family Enrichment

It is often mistakenly believed that “work–family conflict” and “work–family balance” are opposite concepts. However, the opposite of “work–family balance” is not “work–family conflict” but rather “work–family imbalance.” “Work–family imbalance” is divided into two categories: positive and negative imbalance. Positive imbalance involves “work–family enrichment,” while the negative imbalance involves “work–family conflict” [24]. The definition of work–family conflict that is generally accepted and adopted by researchers was first proposed by Greenhaus and Beutell in 1985. They defined it as “a form of inter-role conflict in which the role pressures from the work and family domains are mutually incompatible in some respect” [25] (p. 77), and believed that work–family conflict consists of three dimensions: time-based conflict, strain-based conflict and behavior-based conflict [26]. The corresponding positive work–family relationship is work–family enrichment. In 2006, Greenhaus and Powell defined work–family enrichment as “the extent to which experiences in one role improve the quality of life in another role” and argued that work–family enrichment can be generated through two paths, namely, the instrumental path and the affective path [27].

According to the authors’ literature review on the studies related to work–family relationships in CPMPs [24], most studies have focused on work–family conflict and its antecedent [28,29,30,31] and outcome variables [22]. Compared to work–family conflict, limited studies have focused on work–family enrichment [32]. This situation indicates that work–family conflict is prominent among CPMPs, which is generally recognized by scholars; in contrast, prior studies lack a comprehensive, in-depth understanding of the complex mechanism between the work and family domains and tend to focus on negative phenomena while ignoring the potentially positive effects of work–family enrichment.

### 2.2. Research on Job Burnout and Work Engagement

Job burnout and work engagement are widely used to characterize professionals’ long-term occupational mental health. Maslach identified job burnout as “a psychological syndrome that arises in response to chronic interpersonal stress at work [33].” It has three dimensions: exhaustion, in which the individual’s resources are excessively consumed, representing the stress aspect of job burnout; cynicism, in which the individual reacts negatively, callously, or too superciliously to all aspects of work, representing the interpersonal environment facet of job burnout; and low professional efficacy, which refers to the individual’s feelings of incompetence, lack of achievement and productivity at work, and represents the self-assessment element of job burnout [33].

For a long time, scholars have paid most attention to the negative consequences of stress and burnout to practitioners while ignoring the possible impact of positive psychological qualities. With the development of positive psychology, the concept of work engagement emerged. Schaufeli defined “work engagement” as a positive, enjoyable work-related continuous mental state, comprising three dimensions: vigor, dedication, and absorption [8]. Work engagement is negatively related to job burnout, but it is not entirely opposed. Scholars also found that work engagement could play a significant role in boosting employees’ job satisfaction and decreasing turnover [34].

Our review of the literature shows that most relevant studies in the construction industry have also focused on the negative effects of job stress and job burnout, while less attention was paid to the positive effects of work engagement [7,34]. Thus far, to the best of our knowledge, no study has explored the dual psychological processes of work engagement and job burnout from both negative and positive perspectives in the construction industry at the same time.

### 2.3. Research on FSSB

In 2007, Hammer proposed the concept of FSSB as the informal social support behaviors exhibited by supervisors to support employees’ family roles [19]. FSSB was conceptualized as a trainable, social-support-based resource provided by supervisors to support employees’ ability to fulfill their family responsibilities [7]. This means that family-supportive supervision is no longer just about certain qualities, characteristics, or leadership competencies but is rather a behavioral approach that can be improved through training. Supervisors can help their employees mitigate work–family conflict by practicing emotional support behavior, instrumental support behavior, role modeling behavior, and creative work–family management behavior [35].

Emotional support refers to the supervisor’s willingness to support the employee’s family needs by listening and caring. Instrumental support refers to the supervisor’s willingness to provide targeted support, such as arranging for appropriate time off when responding to the employee’s family needs in daily management. Role modeling behavior refers to the supervisor’s demonstration of how to balance work and family by providing a model at work, such as demonstrating how to be successful both on and off the job. Creative work–family management involves the supervisor’s efforts to help employees balance work and family more effectively by restructuring employees’ work relationships and work content [19,35].

Through the literature review, we found that FSSB proved effective in other industries to help employees alleviate work–family conflict, improve job satisfaction, increase work engagement, and thus, enhance employee performance [36]. A study on Chinese construction workers indicated that FSSB buffered the negative relationship between work–family conflict and safety participation via work engagement [7]. However, the role of FSSB in work–family relationships and occupational mental health for CPMPs deserves more discussion.

## 3. Theoretical Analyses and Hypotheses

### 3.1. Theoretical Base

Scholars have proposed many theoretical models revealing causal relationships related to occupational mental health. Widely used models include the job demand–control (JD-C) model [37], the job demand–control–support (JD-C-S) model [38], the effort–reward imbalance (ERI) model [39], and the job demand–resource (JD-R) model [40].

The first three mainly explain occupational mental health from a negative psychology perspective [41]. The JD-R model argues that there are two independent psychological processes, namely, the health impairment process and the motivational process [42,43]. High job demands lead to strain and health impairment (job burnout), while high job resources lead to increased motivation and higher productivity (work engagement). Based on the JD-R model, Bakker and Geurts further proposed the work–home interface (WHI) model, which suggests that high job demands lead to negative WHI (conflict) and high job resources lead to positive WHI (enrichment) [44]. Integrating the JD-R and WHI models, the authors can develop a positive–negative dual-process psychological model to reflect the actual interwoven psychological process comprehensively for CPMPs.

### 3.2. Relationship between Work–Family Conflict, Work–Family Enrichment, Job Burnout, and Work Engagement

An empirical study on female workers indicated that the stress associated with work–family conflict depletes employees’ emotional resources and prevents individuals from achieving optimal concentration [45], thereby reducing energy and positive emotions at work, which results in lower levels of work engagement. Fiksenbaum indicated that if companies can implement flexible work arrangements and provide employees with more freedom, employees can use their time to lower work–family conflict, which can help improve work engagement [46]. A study on frontline service workers in the hospitality and tourism sectors demonstrated that work–family conflict decreases satisfaction, belonging, and loyalty to the organization, thus, decreasing the individual’s willingness to contribute and commit to the organization [47]. As such, we formulated the following hypothesis:

**H1.**  *Work–family conflict is negatively related to work engagement*.

A significant, positive effect of work–family conflict on job burnout among employees was found in the banking sector [48]. Bruck found that work–family conflict has negative consequences for professionals’ work and family life, physical and mental health, and well-being [49]. This type of conflict increases professionals’ work stress and triggers job burnout [50]. A survey among engineers working in the Australian construction industry indicated that work–family conflict causes mood swings in individuals and may trigger exhaustion or cynicism in employees’ personalities, leading to job burnout [51]. Pinto et al. also concluded that heavy work demands force construction professionals to spend almost all of their energy at work, hence, lack of time with family will exacerbate the conflict between work and family, increasing job burnout [5,41]. Thus, we formulated the following hypothesis:

**H2.**  *Work–family conflict is positively associated with job burnout*.

There are limited studies on the relationship between work–family enrichment and work engagement across industries, suggesting a general lack of attention to the role of positive psychological facilitation. Qing and Zhou found that when employees experience work–family enrichment, they are likely to attribute better family functioning to benefits stemming from their work, which, in turn, facilitates more positive affect and behavioral efforts toward their work to reciprocate the gains achieved from work [52]. It may be that when individuals make attributions about the benefits of one role to another, this primarily results in more positive affect and behavioral investment in the role seen as providing benefit [53]. Siu found that employees were more engaged when they sensed more work–family positive effects [54]. Cinamon and Rich showed that teachers who perceived more work–family enrichment exhibited greater vigor at work [55]. Therefore, we came up with the following hypothesis:

**H3.**  *Work–family enrichment is positively related to work engagement*.

Work–family enrichment may trigger a resource gain spiral, generating a surplus of mental and physical resources, thus, compensating for the potential losses experienced in demanding working conditions [56]. Learning new skills at work and transferring them to one’s family roles (i.e., work-to-family enrichment) may protect individuals from emotional exhaustion and cynicism [57]. Likewise, the family may provide individuals with resources, such as esteem, social support, opportunities for self-growth, and flexibility, that may help them to perform better at work (i.e., family-to-work enrichment) [58]. A study on Chinese knowledge workers found that work–family enrichment was negatively related to job burnout [59], because employees’ overall psychological condition was more positive and confident, hence, they could deal with various difficulties at work and were less likely to develop burnout. Therefore, we developed the following hypothesis:

**H4.**  *Work–family enrichment is negatively related to job burnout*.

### 3.3. Relationship between FSSB and Each Variable

According to the meaning of FSSB, the purpose of FSSB is an intervention approach to alleviate employees’ work–family conflict [60]. Kossek and Crain found FSSB is more effective in mitigating work–family conflict among employees than general supervisory support [61,62]. Further, work–family conflict consumes employees’ emotions, energy, time, and other resources, while FSSB can provide additional psychological resources to alleviate this conflict [63]. Hence, we formulated the following hypothesis:

**H5.**  *FSSB is negatively related to work–family conflict*.

Most studies indicate a positive relationship between FSSB and work–family enrichment. Chan argued that because emotions play an important role in the work–family interface, the emotional care provided by family-supportive supervisors can help professionals have a more energetic mental state to deal with the balance between work and family [64]. Bond and Bunce also found that FSSB can enhance professionals’ career and life satisfaction, promoting work–family enrichment [65]. Thus, we proposed the following hypothesis:

**H6.**  *FSSB is positively related to work–family enrichment*.

Empirical research demonstrated a significant, positive relationship between FSSB and work engagement [66]. Johnson and Matthews explored how FSSB influences positive work- and health-related outcomes, which suggested that FSSB could trigger greater work engagement through an enrichment spiral model [67]. Shi found that social relationship resources (the supervisor-employee exchange relationship) play a mediating role in the relationship between FSSB and work engagement [68]. The work resources provided by supervisors enhance supervisor–employee relationship resources by making employees willing to give some reward to the organization, which further promotes the individual resource input of employees. Therefore, we assumed that CPMPs would exhibit the following relationship:

**H7.**  *FSSB is positively related to work engagement*.

FSSB can help employees to innovatively manage work–family relationships and improve their working style more efficiently. This can give employees more substantial assistance and, thus, increase their satisfaction with the organization, effectively reducing job burnout [69]. When supervisors give employees specific care and understanding, it is equivalent to giving them particular emotional resources, which can reduce professionals’ stress and alleviate job burnout [58]. Yragui found that a supportive family organizational atmosphere allows employees to feel supported and understood and improves their perceptions of work–family relationships. In such an atmosphere, employees can learn how to work efficiently, boost their performance, and successfully alleviate job burnout [70]. Therefore, we hypothesized the following relationship for CPMPs:

**H8.**  *FSSB is negatively related to job burnout*.

### 3.4. Development of the Hypothetical Model

**Figure 1:**
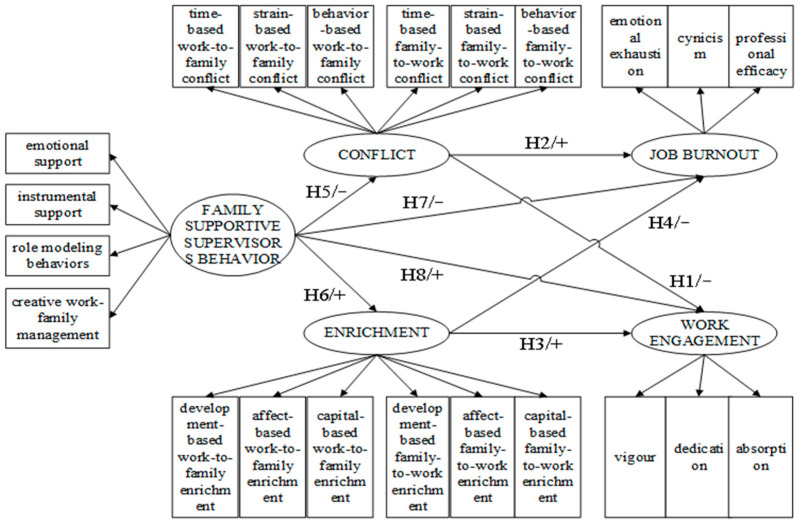
Hypothetical model.

Grounded in the theoretical base and the above hypotheses, we developed a hypothetical model, as shown in Figure 1. The model covers 27 variables, including 5 latent variables, 22 observable variables, and 30 paths. Among them, 8 are the structural paths, and 22 are the measurement paths.

## 4. Materials and Methods

### 4.1. Measures

We adopted widely-used and mature scales to measure the observable variables as shown in Table 1.

### 4.2. Questionnaire Survey

We conducted a web-based questionnaire survey to obtain first-hand data for CPMPs. Each questionnaire was distributed with a copy of a plain-language statement describing the objectives of the study. The statement also explained the voluntary nature of the respondents’ participation and ensured the anonymity of the respondents and the confidentiality of their responses. Finally, the study design was approved by the appropriate ethics review board.

The questionnaire was distributed by targeted distribution to ensure that the intended study population filled out the questionnaire. We sent the web-based questionnaire to China State Construction Engineering Corporation, China Railway Group Limited, China Communications Construction Company Limited, and other large construction engineering contractors. We also used the snowballing method, that is, asking targeted respondents to provide their colleagues and partners as additional respondents, to increase the number of questionnaires returned. The link to the questionnaire was only provided to Chinese construction organizations to ensure that the population studied included only employees of construction enterprises with project site work experience. The survey samples were collected from north, northwest, southwest, southeast, and east China, covering representative provinces with different levels of economic development.

Because it is difficult to control the quality of answers in online questionnaires, the following manual selection standards were set for the returned questionnaires: (1) the questionnaire should take no less than 5 min to complete; (2) the same selection should not be made consecutively in scales; (3) the type of enterprise to which the questionnaires belonged should be checked, and questionnaires for those who chose real estate and other industries should be excluded. A total of 656 questionnaires were returned, 446 were considered valid, representing an effective rate of 70.0%. The descriptive statistics are displayed in Table 2. It can be seen from Table 2 that the samples cover different genders, different age groups, different education levels, different income levels, different positions, and different enterprise holdings, and their distribution characteristics are consistent with the characteristics of industry practitioners, which, to some extent, indicates that the samples are highly representative and reliable.

## 5. Results

### 5.1. Statistics

According to the method proposed by Maslach [74], this study used the average item score for each subscale to measure each dimension of the five latent variables. To observe the distribution pattern of these variables in the sample group, we categorized the statistics according to age, gender, annual personal income, education level, and position level.

**Figure 2:**
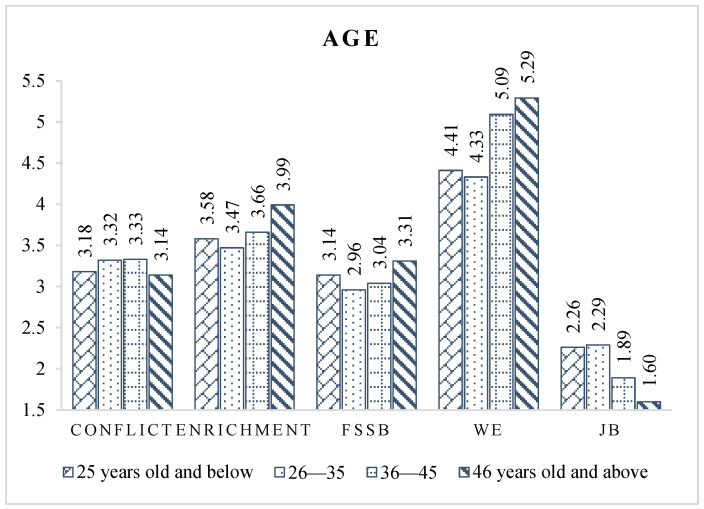
Latent variable scores of different age groups.

By observing the different age groups (Figure 2), we found that people over 46 years had higher ratings for positive latent variables (e.g., work–family enrichment, work engagement, FSSB) and lower ratings for negative variables (work–family conflict, job burnout) than those younger than 46. This was consistent with the findings of professional civil engineers working in the Australian construction industry [75]. This may be because CPMPs are more skilled and better at reconciling work–family relationships after reaching a certain age. It is also possible that individuals and family members are used to and thoroughly adapted to this state of work–family conflict.

The most prominent psychological problems exist in the 26–35 age group, as indicated by the highest levels of conflict and burnout and the lowest levels of enrichment, engagement, and FSSB in the sample population. This age group are the front-line professionals of construction companies, who are also the mainstay and the future of the company. Their prominent psychological problems may originate from the pressure of job promotions, the vast burden brought about by newly formed families, and the lack of sufficient attention and support from senior supervisors [76]. This outcome suggests that construction enterprises should focus on the psychological problems of young professionals in this age group to reduce the loss of excellent talent in the construction industry.

**Figure 3:**
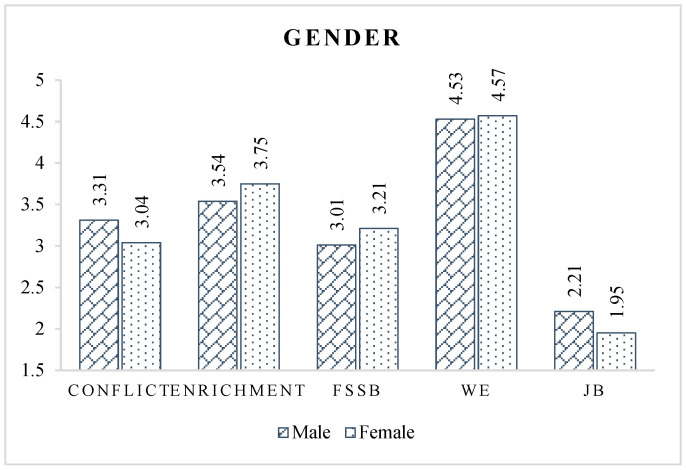
Latent variable scores of different gender groups.

As for gender, Figure 3 implies that women have better overall occupational psychological states and work–family relationships than men. Compared to male professionals, female CPMPs have more regular working hours and more fixed work locations, so the average work stress is low [28]. This may be because most of the positions held by women in the construction industry are office positions that do not require them to be stationed at construction sites for long periods of time.

**Figure 4:**
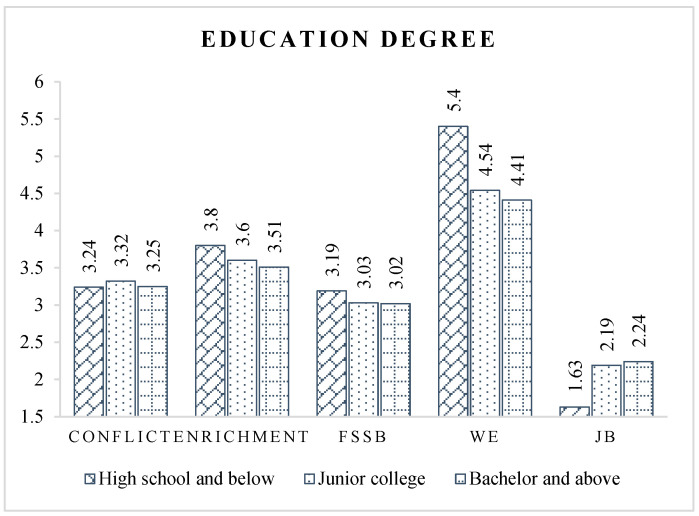
Latent variable scores of different education degree groups.

For education level (Figure 4), CPMPs who completed high school or less have significantly better work–family relationships and occupational psychological status than those with higher education. Compared with those with lower education, those with higher education have higher expectations regarding job content, work environment, income, and personal achievements, increasing the degree of difficulty in achieving expectations, which leads to worse occupational psychological status [33,77].

**Figure 5:**
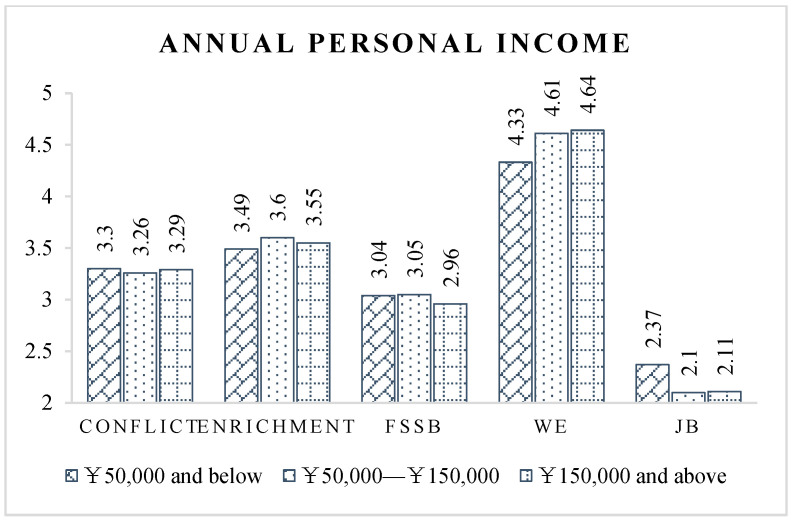
Latent variable scores of different income groups.

As for annual personal income (Figure 5), CPMPs with an annual income of ¥50,000 and below have significantly worse work–family relationships and occupational psychological status than those with higher income. This may be due to the mismatch between these professionals’ high-intensity and high-stress work effort and low incomes. Furthermore, the income they obtain from work is not enough to support their family’s needs, which increases work–family conflict.

**Figure 6:**
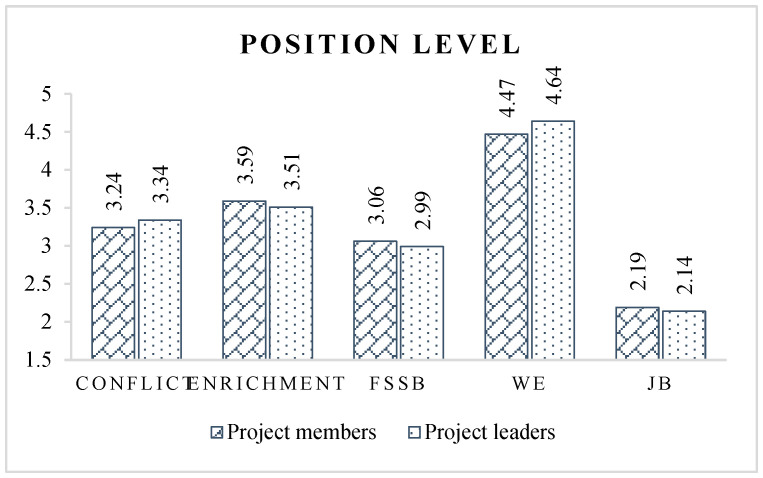
Latent variable scores of different position level groups.

Concerning the position level (Figure 6), the occupational psychological state of project leaders is better than that of project members, showing a higher level of work engagement and a lower level of job burnout. This may be because the work performed by project leaders comprises mostly strategic management and coordination work, giving them a greater sense of achievement and value and motivating them to be more engaged in their work, thus, reducing their burnout level. On the contrary, the work carried out by project members is more trivial, boring, and monotonous, which affects their passion.

However, project leaders have more prominent work–family relationship problems than project members, represented by a greater level of work–family conflict, a lower level of work–family enrichment, together with a lower level of FSSB. The reason may be that project leaders devote more resources to fulfilling their job responsibilities than to their family responsibilities. This result means that for different positions of CPMPs, the psychological issues that construction companies need to pay attention to will also be different.

**Figure 7:**
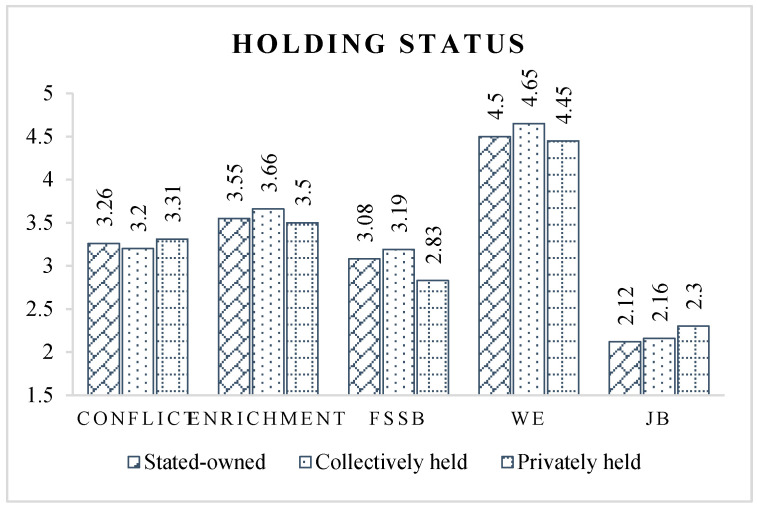
Latent variable scores of different holding status groups.

For the holding status of enterprise (Figure 7), we found that the work–family relationship and occupational psychological state of CPMPs in privately held enterprises are significantly worse than those in collective and state-owned enterprises. It is generally believed that employees of privately held enterprises tend to work under greater stress than those in non-privately held enterprises [2]. CPMPs’ income in privately held companies is often strongly related to work performance. Moreover, in privately held enterprises, CPMPs have more responsibility and the punishment of making mistakes is greater, so their work stress is significantly higher than in non-privately held enterprises [29]. This high-stress environment directly affects CPMPs’ work–family relationships and occupational psychology, as evidenced by greater levels of work–family conflict and job burnout, and lower levels of work–family enrichment and work engagement.

### 5.2. Reliability and Validity

This study mainly used SPSS 22.0 and AMOS 24.0 software for analysis. SPSS 22.0 was used for descriptive statistical analysis of variables, one-way analysis of variance, exploratory factor analysis, and bivariate correlation analysis. AMOS 24.0 was used to conduct the validation factor analysis of the scale and the structure equation modeling analysis.

The method used to estimate the parameters of this model was the maximum likelihood estimation. We used the Cronbach’s alpha coefficient to evaluate the internal consistency of the scales; the internal consistency of all sub-scales and scales was acceptable (>0.6) and good (>0.8), respectively (Table 3).

χ^2^/df, root mean square error of approximation (RMSEA), goodness-of-fit index (GFI), non-normed fit index (NNFI), comparative fit index (CFI), adjusted goodness-of-fit Index (AGFI), and incremental fit index (IFI) are commonly used indicators to test the fitness of the model.

For the fitting indicators (Table 4), χ^2^/df < 5, RMSEA < 0.08, GFI, NNFI, and CFI were basically above 0.9 and close to 1. AGFI and IFI were also close to 1, which indicated that the fit was acceptable [78]. Therefore, the preliminary judgment is that all five scales have good validity.

Convergent validity tests (Table 5) showed that the factor loadings of all items were greater than 0.5, CR > 0.7, and the AVE was basically greater than 0.5, indicating that the scale had acceptable structural validity [79].

### 5.3. Structural Equation Model

**Figure 8:**
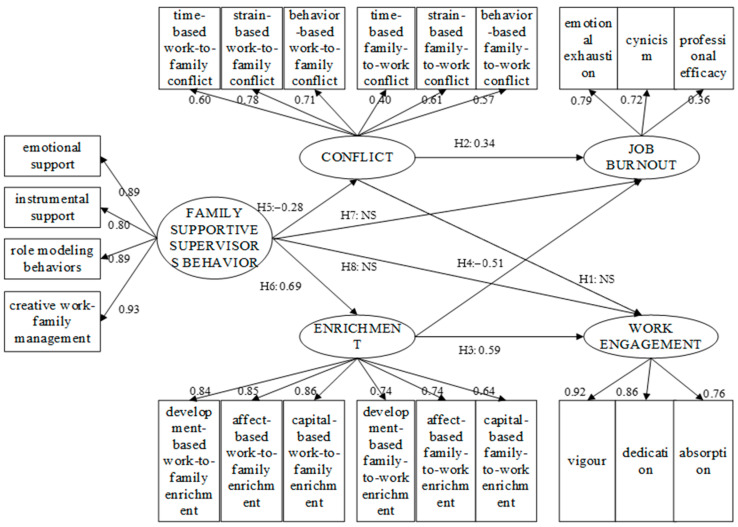
Final accepted model.

We used SPSS AMOS for the linear covariance equation regression. The results showed that most hypotheses were verified, except for the paths of H1, H7, and H8. After modifying our structural equation model (Figure 8), the fitness was finally acceptable (Table 6).

## 6. Discussion

### 6.1. The Relationships among Work–Family Conflict, Work–Family Enrichment, Job Burnout, and Work Engagement

By observing the structural model, we found that work–family enrichment was significantly associated with both job burnout and work engagement. This outcome suggests that the hypothetical effects of work–family enrichment on job burnout and work engagement are valid. Work–family enrichment can enhance CPMPs’ physical and emotional resources through the emotional path and improve personnel’s abilities through the instrumental path, thus, effectively deepening personnel’s work engagement and reducing job burnout. This is consistent with the findings of Robinson [57].

While based on the outcomes of previous studies in the construction industry, our original expectation was that work–family conflict would play a dominant role in the final accepted model. However, the outcomes demonstrate that the hypotheses on the role of work–family enrichment hold in all cases, while there is a non-significant path for work–family conflict (H1). Work–family conflict has significant positive effect on job burnout, which means that CPMPs with higher level of work–family conflict are more likely to experience job burnout. This significant path is consistent with prior research on the construction industry, reflecting that the depletion of physical and emotional resources triggered by work–family conflict exacerbates job burnout among CPMPs.

More importantly, the results showed that work–family enrichment had a more significant effect on the occupational mental health status of CPMPs than work–family conflict. Moreover, we can see that work–family enrichment also plays a more dominant role than work–family conflict by comparing the magnitude of the path coefficients. The reason for this may be that the inherent characteristics of construction projects make work–family conflict in the construction sector inevitable, which leads to a diminished effect of work–family conflict on the psychological state of work so that work–family conflict does not have an imaginary dominant effect. On the contrary, if some positive psychological factors (e.g., work–family enrichment, FSSB) are present in this negative state, CPMPs and their families will be more sensitive to such good aspects. Thus, the positive psychological factors play a more prominent role. This inference can also be supported by examining the mean score of work–family enrichment and conflict. The mean score for work–family enrichment (3.58/5) was greater than the mean score for work–family conflict (3.28/5) for the surveyed sample responses, indicating that CPMPs are more empathetic to the role of work–family enrichment, while work–family conflict is not as prominently felt as we might expect.

This finding has important theoretical and practical implications for construction management. Theoretically, the study confirms the basic assumption of positive psychology: CPMPs determine their subsequent actions with consideration of promising visions and expectations for the future, rather than only being driven by painful experiences that have already occurred in the past. Therefore, future research should pay attention to the positive psychology process along with the negative one, which can provide new perspectives and guarantees for a comprehensive understanding of CPMPs’ complex occupational psychological issues. In practice, the results suggest that adjusting CPMPs’ work engagement and job burnout by mitigating work–family conflict in construction enterprises is not the most effective way. In contrast, supervisors can enhance CPMPs’ resources by stimulating work–family enrichment’s instrumental and emotional role to effectively improve their occupational psychological health.

### 6.2. The Relationship between FSSB and Each Variable

The structural model reveals that the FSSB is significantly related to work–family conflict and enrichment. This finding indicates that CPMPs’ work–family issues have begun to be taken by construction enterprises and project supervisors, and certain supportive behaviors were gradually adopted to reduce their work–family conflict and enhance work–family enrichment. The results imply that supervisors should care about CPMPs’ family needs proactively, help them relieve family stress, and develop innovative family-supportive policies to promote their work–family enrichment. Furthermore, scholars also began to take FSSB as an intervention tool to improve employees’ work–family relationship [80]. A new study demonstrated that employees’ reaction to FSSB depends upon role beliefs [81]. Accordingly, construction enterprises can design intervention plans in conjunction with the reality of the enterprise. For instance, enterprises can regularly give CPMPs work–family benefit vouchers to help them balance work and family. A family-supportive atmosphere can make CPMPs who are in a chronic high-pressure environment feel understood and supported while improving their perception of work–family relationships. Furthermore, supervisors can also focus on enhancing their own ability to balance work and family to serve as an example to their professionals.

However, we found no significant correlation between FSSB and job burnout or work engagement. In other words, work–family conflict and enrichment played a fully mediating role in the relationship between FSSB and job burnout and work engagement. Existing research has consistently shown that informal workplace support, such as FSSB, is more effective at reducing work–family conflict than formal organizational supports [81]. Although it is difficult to directly influence professionals’ occupational mental health, enterprises and supervisors should not neglect CPMPs’ work–family relationships and should use refined management tools such as FSSB. In the long term, FSSB can effectively improve CPMPs’ work–family relationships and, thus, indirectly enhance their occupational mental health status.

## 7. Conclusions

Most current studies on the mental health of CPMPs were conducted from a negative psychological perspective, lacking a comprehensive understanding of the positive–negative dual-process mechanism and neglecting the supportive role of FSSB. In this study, we proposed a model integrating the positive–negative dual-process mechanism of the mental health of CPMPs to explore the interwoven mechanisms among five variables: FSSB, work–family conflict, work–family enrichment, job burnout, and work engagement. We conducted a large-scale questionnaire survey based on widely-used and mature scales, and statistically analyzed the distribution patterns of the five variables rooted in the demographic differences in the survey sample. We concluded the study by applying structural equation modeling to verify the proposed hypothetical model and to provide an in-depth analysis of the possible reasons behind the significant paths.

We found that the occupational psychological state of those who are older and have many years of experience is better, whereas the occupational psychological problems of young professionals aged 26–35 are most prominent; the occupational psychological state and work–family relationships of women are generally better than those of men; the occupational psychological state of those with a low education level is better than those with a high education level; and the occupational psychological state of those with higher annual personal incomes is better. These outcomes clarify the groups of CPMPs prone to occupational psychological problems. It is of great significance for enhancing the targeted and effective organizational management in the construction industry.

Compared with work–family conflict, the effect of work–family enrichment on CPMPs’ occupational psychological health status is more significant. This finding has important theoretical and practical implications for construction management. Theoretically, the results imply that the positive psychology process may play a more prominent role than the traditional negative one; hence, it needs to be paid enough attention [82]. Therefore, future research may introduce more tools from positive psychology to complement the traditional negative psychological processes, which will provide new perspectives and guarantees for a thorough understanding of the complex occupational psychological problems of construction professionals. Empirically, the results show that traditional management methods for CPMPs’ work engagement and job burnout through regulating their work–family conflict in construction enterprises is not the most effective way. Supervisors can try to increase the level of individual resources by stimulating the tool and the emotion roles of work–family enrichment to effectively improve CPMPs’ occupational psychological health.

There is a significant correlation between FSSB and work–family conflict and enrichment, indicating that the current work–family problems of CPMPs have begun to be taken seriously by enterprises and project supervisors. They have gradually adopted certain supportive behaviors to reduce work–family conflict and boost the enrichment of CPMPs. However, there is no significant correlation between FSSB and the aspects of long-term occupational psychological health, that is, job burnout and work engagement. This indicates that work–family conflict and enrichment played a fully mediating role in the relationship between FSSB and job burnout and work engagement.

There are limitations to this study, and more in-depth research is needed in the following areas: (1) The data obtained in this study are cross-sectional web data, and long-term observations are needed to further verify the hypothetical model with more reliable panel data; (2) This study provides a theoretical framework, and the FSSB interventions should be implemented to verify the credibility and feasibility of the mechanisms concluded in this study; (3) This study provides a group-specific analysis in the Chinese construction context, and owing to the disadvantages of snowball sampling, the age distribution of respondents was uneven; therefore, the applicability of the theoretical framework needs to be further verified in different groups.

## Figures and Tables

**Table 1:** Scales for questionnaire.

Latent Variables	Scales	Measurement Dimensions (Observable Variables)
Work–family conflict (WFC)	18-item, 5-point Likert scale [71]	Time-based work-to-family conflict (WFT)
		Strain-based work-to-family conflict (WFS)
		Behavior-based work-to-family conflict (WFB)
		Time-based family-to-work conflict (FWT)
		Strain-based family-to-work conflict (FWS)
		Behavior-based family-to-work conflict (FWB)
Work–family enrichment (WFE)	18-item, 5-point Likert scale [72]	Development-based work-to-family enrichment (WFD)
		Affect-based work-to-family enrichment (WFA)
		Capital-based work-to-family enrichment (WFCa)
		Development-based family-to-work enrichment (FWD)
		Affect-based family-to-work enrichment (FWA)
		Capital-based family-to-work enrichment (FWCa)
Job burnout (JB)	MBI-GS, 16-item, 7-point Likert scale [73]	Exhaustion (EX)
		Cynicism (CY)
		Low professional efficacy (PE)
Work engagement (WE)	Short-version UWES, 9-item, 7-point Likert scale [74]	Vigor (VI)
		Dedication (DE)
		Absorption (AB)
FSSB	14-item, 5-point Likert scale [35]	Emotional support (ES)
		Instrumental support (IS)
		Role modeling (RM)
		Creative management (CM)

**Table 2:** Demographic distribution of the sample data.

Dimensions	Group	Frequency	Proportion	Cumulative Percentage
Gender	Male	390	87.44%	87.44%
	Female	56	12.56%	100.00%
Age	25 years old and below	102	22.87%	22.87%
	26–30 years old	160	35.87%	58.74%
	31–35 years old	87	19.51%	78.25%
	36–40 years old	43	9.64%	87.89%
	41–45 years old	21	4.71%	92.60%
	46–50 years old	21	4.71%	97.31%
	51 years old and above	12	2.69%	100.00%
Education level	High school and below	34	7.62%	7.62%
	Junior college	161	36.10%	43.72%
	Bachelor	245	54.93%	98.65%
	Master	6	1.35%	100.00%
Income	¥50,000 and below	127	28.48%	28.48%
	¥50,000–¥150,000	276	61.88%	90.36%
	¥150,000–¥300,000	33	7.40%	97.76%
	¥300,000–¥500,000	8	1.79%	99.55%
	¥500,000 and above	2	0.45%	100.00%
Position level	Project members	284	63.68%	63.68%
	Project leaders	162	36.32%	100.00%
Holdings	State-owned	192	43.05%	43.05%
	Collective	81	18.16%	61.21%
	Privately held	124	27.80%	89.01%
	Unclear	49	10.99%	100.00%

**Table 3:** Internal consistency.

Scale	Sub-Scale	Alpha of Sub-Scale	Alpha of Scale
WFC	WFT	0.694	0.854
	WFS	0.661	
	WFB	0.637	
	FWT	0.700	
	FWS	0.669	
	FWB	0.651	
WFE	WFD	0.858	0.946
	WFA	0.863	
	WFCa	0.898	
	FWD	0.835	
	FWA	0.853	
	FWCa	0.791	
FSSB	ES	0.902	0.959
	IS	0.829	
	RM	0.903	
	CM	0.911	
WE	VI	0.891	0.925
	DE	0.858	
	AB	0.818	
JB	EX	0.875	0.871
	CY	0.801	
	PE	0.843	

**Table 4:** Fitting indicators of structural equation model.

Scale	χ^2^/df	RMSEA	GFI	NNFI	CFI	AGFI	IFI
WFC	2.785	0.063	0.921	0.876	0.903	0.888	0.904
WFE	3.453	0.074	0.896	0.932	0.947	0.851	0.947
FSSB	2.487	0.058	0.947	0.974	0.980	0.922	0.980
WE	3.948	0.080	0.956	0.961	0.974	0.917	0.974
JB	3.246	0.071	0.914	0.916	0.929	0.885	0.930

**Table 5:** Convergent validity.

Observable Variable	Items	Estimates	CR	AVE
WFT	WFT1	0.779	0.768	0.526
	WFT2	0.677		
	WFT3	0.716		
WFS	WFS1	0.691	0.745	0.495
	WFS2	0.632		
	WFS3	0.779		
WFB	WFB1	0.715	0.751	0.502
	WFB2	0.695		
	WFB3	0.715		
FWT	FWT1	0.726	0.773	0.531
	FWT2	0.722		
	FWT3	0.738		
FWS	FWS1	0.603	0.747	0.501
	FWS2	0.673		
	FWS3	0.829		
FWB	FWB1	0.716	0.746	0.496
	FWB2	0.637		
	FWB3	0.755		
WFD	WFD1	0.748	0.859	0.670
	WFD2	0.855		
	WFD3	0.848		
WFA	WFA1	0.774	0.855	0.663
	WFA2	0.833		
	WFA3	0.872		
WFCa	WFCa1	0.897	0.899	0.748
	WFCa2	0.870		
	WFCa3	0.826		
FWD	FWD1	0.762	0.835	0.629
	FWD2	0.815		
	FWD3	0.801		
FWA	FWA1	0.829	0.853	0.660
	FWA2	0.789		
	FWA3	0.818		
FWCa	FWCa1	0.676	0.792	0.563
	FWCa2	0.688		
	FWCa3	0.871		
ES	ES1	0.835	0.902	0.698
	ES2	0.855		
	ES3	0.794		
	ES4	0.856		
IS	IS1	0.736	0.829	0.620
	IS2	0.765		
	IS3	0.853		
RM	RM1	0.852	0.900	0.748
	RM2	0.890		
	RM3	0.866		
CM	CM1	0.825	0.911	0.720
	CM2	0.859		
	CM3	0.832		
	CM4	0.874		
VI	VI1	0.875	0.892	0.733
	VI2	0.859		
	VI3	0.834		
DE	DE1	0.870	0.863	0.677
	DE2	0.801		
	DE3	0.796		
AB	AB1	0.786	0.822	0.606
	AB2	0.730		
	AB3	0.818		
EX	EX1	0.835	0.876	0.586
	EX2	0.682		
	EX3	0.738		
	EX4	0.781		
	EX5	0.784		
CY	CY1	0.596	0.825	0.492
	CY2	0.526		
	CY3	0.748		
	CY4	0.782		
	CY5	0.811		
PE	PE1	0.769	0.857	0.502
	PE2	0.697		
	PE3	0.645		
	PE4	0.667		
	PE5	0.806		
	PE6	0.652		

**Table 6:** The fitness of the structural equation modeling.

χ^2^/df	RMSEA	GFI	NNFI	CFI	AGFI	IFI
3.325	0.068	0.907	0.914	0.922	0.901	0.923

## Data Availability

Some or all data, models, or code that support the findings of this study are available from the corresponding author upon reasonable request.

## References

[1] Yang F., Li X., Song Z., Li Y., Zhu Y. (2018). Job burnout of construction project managers: Considering the role of organizational justice. J. Constr. Eng. Manag..

[2] Yang F., Li X., Zhu Y., Li Y., Wu C. (2017). Job burnout of construction project managers in China: A cross-sectional analysis. Int. J. Proj. Manag..

[3] Holden S., Sunindijo R.Y. (2018). Technology, long work hours, and stress worsen work-life balance in the construction industry. Int. J. Integr. Eng..

[4] Campbell F. (2006). Occupational Stress in the Construction Industry.

[5] Pinto J.K., Patanakul P., Pinto M.B. (2016). Project personnel, job demands, and workplace burnout: The differential effects of job title and project type. IEEE Trans. Eng. Manag..

[6] Yip B., Rowlinson S. (2009). Job burnout among construction engineers working within consulting and contracting organizations. J. Manag. Eng..

[7] Xie Q., Xia N., Yang G. (2022). Do family affairs matter? Work–family conflict and safety behavior of construction workers. J. Manag. Eng..

[8] Schaufeli W.B., Salanova M., González-Romá V., Bakker A.B. (2002). The measurement of engagement and burnout: A two sample confirmatory factor analytic approach. J. Happiness Stud..

[9] Seligman M.E.P., Csikszentmihalyi M. (2000). Positive psychology: An introduction. Am. Psychol..

[10] Gable S.L., Haidt J. (2005). What (and why) is positive psychology?. Rev. Gen. Psychol..

[11] Peterson C. (2009). Positive psychology. Reclaiming Child. Youth.

[12] Russell J.A., Carroll J.M. (1999). On the bipolarity of positive and negative affect. Psychol. Bull..

[13] Schaufeli W.B., Bakker A.B. (2004). Job demands, job resources, and their relationship with burnout and engagement: A multi-sample study. J. Organ. Behav..

[14] Demerouti E., Mostert K., Bakker A.B. (2010). Burnout and work engagement: A thorough investigation of the independency of both constructs. J. Occup. Health Psychol..

[15] George J.M. (2011). Dual tuning. Organ. Psychol. Rev..

[16] Dulk L.D., de Ruijter J. (2008). Managing work-life policies: Disruption versus dependency arguments. Explaining managerial attitudes towards employee utilization of work-life policies. Int. J. Hum. Resour. Manag..

[17] McCarthy A., Darcy C., Grady G. (2010). Work-life balance policy and practice: Understanding line manager attitudes and behaviors. Hum. Resour. Manag. Rev..

[18] Straub C. (2011). Antecedents and organizational consequences of family supportive supervisor behavior: A multilevel conceptual framework for research. Hum. Resour. Manag. Rev..

[19] Hammer L.B., Kossek E.E., Zimmerman K., Daniels R., Perrewe P.L., Ganster D.C. (2007). Clarifying the construct of family-supportive supervisory behaviors (FSSB): A multilevel perspective. Exploring the Work and Non-Work Interface.

[20] Crain T.L., Stevens S.C. (2018). Family-supportive supervisor behaviors: A review and recommendations for research and practice. J. Organ. Behav..

[21] Leung M., Chan Y.S.I., Dongyu C. (2011). Structural linear relationships between job stress, burnout, physiological stress, and performance of construction project managers. Eng. Constr. Arch. Manag..

[22] Chan I.Y.S., Leung M.-Y., Liang Q. (2018). The roles of motivation and coping behaviours in managing stress: Qualitative interview study of Hong Kong expatriate construction professionals in mainland China. Int. J. Environ. Res. Public Health.

[23] Odle-Dusseau H.N., Britt T.W., Greene-Shortridge T.M. (2012). Organizational work–family resources as predictors of job performance and attitudes: The process of work–family conflict and enrichment. J. Occup. Health Psychol..

[24] Wang X.Y., Li X.D., Yang F., Zhang Z.H. (2019). Review on work-family relationships of construction professionals. J. Eng. Manag..

[25] Greenhaus J.H., Beutell N.J. (1985). Sources of conflict between work and family roles. Acad. Manag. Rev..

[26] Carlson D.S. (1999). Personality and role variables as predictors of three forms of work–family conflict. J. Vocat. Behav..

[27] Greenhaus J.H., Powell G.N. (2006). When work and family are allies: A theory of work-family enrichment. Acad. Manag. Rev..

[28] Lingard H., Lin J. (2003). Managing motherhood in the Australian construction industry: Work-family Balance, Parental Leave and Part-time Work. Constr. Econ. Build..

[29] Francis V., Lingard H., Prosser A., Turner M. (2013). Work-family and construction: Public and private sector differences. J. Manag. Eng..

[30] Turner M., Lingard H., Francis V. (2009). Work-life balance: An exploratory study of supports and barriers in a construction project. Int. J. Manag. Proj. Bus..

[31] Davis H., Francis V. (2016). ICT in construction: Can it reduce work-family conflict by decreasing workloads?. Proceedings of the 28th Australian Conference on Computer-Human Interaction, Launceston, Australia, 29 November–2 December 2016.

[32] Lingard H.C., Francis V., Turner M. (2010). Work–family enrichment in the Australian construction industry: Implications for job design. Constr. Manag. Econ..

[33] Maslach C., Schaufeli W.B., Leiter M.P. (2001). Job Burnout. Annu. Rev. Psychol..

[34] Li X., Fei Y., Rizzuto T.E., Yang F. (2020). What are the occupational hazards of construction project managers: A data mining analysis in China. Saf. Sci..

[35] Hammer L.B., Kossek E.E., Yragui N.L., Bodner T.E., Hanson G.C. (2008). Development and validation of a multidimensional measure of family supportive supervisor behaviors (FSSB). J. Manag..

[36] Hammer L.B., Kossek E.E., Anger W.K., Bodner T., Zimmerman K.L. (2011). Clarifying work–family intervention processes: The roles of work–family conflict and family-supportive supervisor behaviors. J. Appl. Psychol..

[37] Karasek R.A. (1979). Job demands, job decision latitude, and mental strain: Implications for job redesign. Adm. Sci. Q..

[38] Johnson J.V., Hall E.M. (1988). Job strain, work place social support, and cardiovascular disease: A cross-sectional study of a random sample of the Swedish working population. Am. J. Public Health.

[39] Siegrist J. (1996). Adverse health effects of high-effort/low-reward conditions. J. Occup. Health Psychol..

[40] Demerouti E., Bakker A.B., Nachreiner F., Schaufeli W.B. (2001). The job demands-resources model of burnout. J. Appl. Psychol..

[41] Pinto J.K., Dawood S., Pinto M.B. (2014). Project management and burnout: Implications of the Demand–Control–Support model on project-based work. Int. J. Proj. Manag..

[42] Bakker A.B., Demerouti E., Verbeke W. (2004). Using the job demands-resources model to predict burnout and performance. Hum. Resour. Manag..

[43] Schaufeli W.B., Bakker A.B., Van Rhenen W. (2009). How changes in job demands and resources predict burnout, work engagement, and sickness absenteeism. J. Organ. Behav..

[44] Bakker A.B., Geurts S.A.E. (2004). Toward a dual-process model of work-home interference. Work Occup..

[45] Purwayoga P.V.S., Dharmanegara I.B.A., Yasa P.N.S. (2019). Mediating role of work engagement and emotional exhaustion in the effect of work-family conflict on female workers’ turnover intention. Int. J. Acad. Res. Bus. Soc. Sci..

[46] Fiksenbaum L.M. (2013). Supportive work–family environments: Implications for work–family conflict and well-being. Int. J. Hum. Resour. Manag..

[47] Mansour S., Tremblay D.-G. (2016). Work–family conflict/family–work conflict, job stress, burnout and intention to leave in the hotel industry in Quebec (Canada): Moderating role of need for family friendly practices as “resource passageways”. Int. J. Hum. Resour. Manag..

[48] Laeeque S.H. (2014). Role of work-family conflict in job burnout: Support from the banking sector of Pakistan. Int. Lett. Soc. Humanist. Sci..

[49] Bruck C.S., Allen T., Spector P.E. (2002). The relation between work–family conflict and job satisfaction: A finer-grained analysis. J. Vocat. Behav..

[50] Shockley K.M., Singla N. (2011). Reconsidering work—Family interactions and satisfaction: A meta-analysis. J. Manag..

[51] Lingard H. (2003). The impact of individual and job characteristics on ‘burnout’ among civil engineers in Australia and the implications for employee turnover. Constr. Manag. Econ..

[52] Qing G., Zhou E., Guoxia Q., Erhua Z. (2017). Bidirectional work–family enrichment mediates the relationship between family-supportive supervisor behaviors and work engagement. Soc. Behav. Pers. Int. J..

[53] Wayne J.H., Musisca N., Fleeson W. (2004). Considering the role of personality in the work–family experience: Relationships of the big five to work–family conflict and facilitation. J. Vocat. Behav..

[54] Siu O.L., Bakker A.B., Brough P., Lu C.-Q., Wang H., Kalliath T., O’Driscoll M., Lu J., Timms C. (2015). A three-wave study of antecedents of work-family enrichment: The roles of social resources and affect. Stress Health.

[55] Cinamon R.G., Rich Y. (2009). Work family relations: Antecedents and outcomes. J. Career Assess..

[56] Innstrand S.T., Langballe E.M., Espnes G.A., Falkum E., Aasland O.G. (2008). Positive and negative work–family interaction and burnout: A longitudinal study of reciprocal relations. Work Stress.

[57] Robinson L.D., Magee C., Caputi P. (2016). Burnout and the work-family interface. Career Dev. Int..

[58] Ollier-Malaterre A., Haar J.M., Sunyer A., Russo M. (2019). Supportive organizations, work–family enrichment, and job burnout in low and high humane orientation cultures. Appl. Psychol..

[59] Xiaobing Z., Shiyun Z. The effects of work-family enrichment on knowledge workers’ job burnout and mental health. Proceedings of the IEEE.

[60] Mostert K. (2011). Job characteristics, work–home interference and burnout: Testing a structural model in the South African context. Int. J. Hum. Resour. Manag..

[61] Kossek E.E., Pichler S., Bodner T., Hammer L.B. (2011). Workplace social support and work-family conflict: A meta-analysis clarifying the influence of general and work-family-specific supervisor and organizational support. Pers. Psychol..

[62] Crain T.L., Hammer L.B., Bodner T., Kossek E.E., Moen P., Lilienthal R., Buxton O.M. (2014). Work–family conflict, family-supportive supervisor behaviors (FSSB), and sleep outcomes. J. Occup. Health Psychol..

[63] Kailasapathy P., Jayakody J.A.S.K. (2016). Does leadership matter? Leadership styles, family supportive supervisor behaviour and work interference with family conflict. Int. J. Hum. Resour. Manag..

[64] Chan X.W., Kalliath P., Chan C., Kalliath T. (2019). How does family support facilitate job satisfaction? Investigating the chain mediating effects of work–family enrichment and job-related well-being. Stress Health.

[65] Bond F.W., Bunce D. (2003). The role of acceptance and job control in mental health, job satisfaction, and work performance. J. Appl. Psychol..

[66] Bakker A.B., Demerouti E. (2007). The Job Demands-Resources model: State of the art. J. Manag. Psychol..

[67] Matthews R.A., Mills M.J., Trout R.C., English L. (2014). Family-supportive supervisor behaviors, work engagement, and subjective well-being: A contextually dependent mediated process. J. Occup. Health Psychol..

[68] Shi Y., Xie J., Zhou Z.E., Tang H., Ma H. (2019). Family supportive supervisor behaviors and work engagement: A social information processing perspective. Curr. Psychol..

[69] Rofcanin Y., Heras M.L., Escribano P.I., Stanko T. (2019). FSSBs and elderly care: Exploring the role of organizational context on employees’ overall health and work–family balance satisfaction. J. Bus. Psychol..

[70] Yragui N.L., Demsky C.A., Hammer L.B., Van Dyck S., Neradilek M.B. (2016). Linking workplace aggression to employee well-being and work: The moderating role of family-supportive supervisor behaviors (FSSB). J. Bus. Psychol..

[71] Carlson D.S., Kacmarb K.M., Williams L.J. (2000). Construction and initial validation of a multidimensional measure of work–family conflict. J. Vocat. Behav..

[72] Carlson D.S., Kacmar K.M., Wayne J.H., Grzywacz J.G. (2006). Measuring the positive side of the work–family interface: Development and validation of a work–family enrichment scale. J. Vocat. Behav..

[73] Maslach C., Jackson S., Leiter M. (1997). The Maslach Burnout Inventory Manual.

[74] Schaufeli W.B., Bakker A.B., Salanova M. (2006). The measurement of work engagement with a short questionnaire: A cross-national study. Educ. Psychol. Meas..

[75] Lingard H., Sublet A. (2002). The impact of job and organizational demands on marital or relationship satisfaction and conflict among Australian civil engineers. Constr. Manag. Econ..

[76] Loughlin C., Barling J. (2001). Young workers’ work values, attitudes, and behaviours. J. Occup. Organ. Psychol..

[77] Mohammadpoorasl A., Maleki A., Sahebihagh M.H. (2012). Prevalence of professional burnout and its related factors among nurses in Tabriz in 2010. Iran. J. Nurs. Midwifery Res..

[78] Browne M.W., Cudeck R. (1992). Alternative ways of assessing model fit. Sociol. Methods Res..

[79] Fornell C., Larcker D.F. (1981). Structural equation models with unobservable variables and measurement error: Algebra and statistics. J. Mark. Res..

[80] Davis K.D., Lawson K.M., Almeida D.M., Kelly E.L., King R.B., Hammer L., Casper L.M., Okechukwu C.A., Hanson G., McHale S.M. (2015). Parents’ daily time with their children: A workplace intervention. Pediatrics.

[81] Yu A., Pichler S., Russo M., Hammer L. (2021). Family-supportive supervisor behaviors (FSSB) and work-family conflict: The role of stereotype content, supervisor gender, and gender role beliefs. J. Occup. Organ. Psychol..

[82] Jiang H., Ma H., Xie J., Zhang S. (2015). The effects of family-supported supervisor behavior on work attitudes: A moderated mediating model. J. Psychol. Sci..

